# Iatrogenic physiologic opioid dependence masquerading as recurrent acute pancreatitis flares: case series

**DOI:** 10.1186/s13722-026-00653-7

**Published:** 2026-02-12

**Authors:** David Dayan-Rosenman, Sara Diletti-Swenson, Nisha Ghayalod, Randi Sokol

**Affiliations:** 1 Cambridge Health Alliance & Harvard Medical School, 1493 Cambridge St., Cambridge, MA 02139 USA; 2 Cambridge Health Alliance, 195 Canal St., Malden, MA 02148 USA; 3 Cambridge Health Alliance & Harvard Medical School, 195 Canal St., Malden, MA 02148 USA

**Keywords:** Iatrogenic physiologic opioid dependence, Pancreatitis, Opioid withdrawal, Recurrent hospitalizations, Buprenorphine, Case series

## Abstract

Patients with recurrent acute flares of pancreatitis—especially those receiving frequent opioid treatment for pain—can develop physiologic dependence to opioids. This can complicate the clinical picture, making it difficult to distinguish between true pancreatitis flares and opioid withdrawal presenting with similar symptoms. We describe two cases of patients admitted recurrently for pancreatitis: a 24-year-old Haitian male with alcohol use disorder, and a 37-year-old Indian female with hypertriglyceridemia. Both developed iatrogenic physiologic opioid dependence after frequent hospitalizations and opioid prescriptions. Both patients stabilized after initiation of buprenorphine, remaining in care and hospitalization-free for over a year. These cases illustrate the importance of identifying and treating the underlying etiology of abdominal pain, judiciously prescribing opioids to treat pancreatitis flares, and having a low threshold for considering iatrogenic physiologic opioid dependence in patients evaluated for recurrent pancreatitis. They also highlight the potential role that buprenorphine can play to mitigate recurrent admissions by providing safer pain control and treatment for physiologic opioid dependence.

## Background

Pancreatitis, both acute and chronic, remains a leading cause of gastrointestinal-related hospitalizations, with incidence increasing since the 1980s [1]. Chronic pancreatitis is particularly debilitating, associated with poor quality of life, high disability rates, and significant opioid use [2]. Despite diverse etiologies — alcohol, biliary, hyperlipidemia, and genetics — pain control remains the cornerstone of treatment, often relying on opioid agonists [3, 4]. 

Chronic use of opioids in these patients is linked to increased hospitalizations, higher rates of alcohol and tobacco use, and higher healthcare utilization [5]. It also increases the risk for developing opioid use disorder (OUD) [6]. Similar to methadone, buprenorphine is an evidence-supported medication for the treatment of OUD [7]. Buprenorphine leads to reduced overdose-related and all-cause mortality rates, decreased risk of return to non-prescribed opioid use, and improved retention in treatment [8, 9]. It is considered a the preferred opioid over full agonist opioids for chronic pain by multiple governmental and non-governmental bodies including the U.S. Department of Veterans Affairs [10, 11], the US Department of Health and Human Services [12], and the American Society of Addiction Medicine [13]. It holds particular promise in patients at higher risk of developing substance use disorders [14, 15]. However, there is a dearth of literature on use of buprenorphine for treatment of physiologic opioid dependence (as distinct from opioid use disorder) in the setting of chronic pancreatitis. In our clinical experience, we have encountered patients hospitalized repeatedly for presumed pancreatitis flares who were later recognized instead to be experiencing episodes of opioid withdrawal. These patients stabilized on buprenorphine, with no subsequent hospitalizations. We propose that opioid withdrawal can mimic acute pancreatitis flares, and that outpatient treatment with buprenorphine may be an effective treatment in this setting.

We have received permission from both patients to discuss their medical course for the purpose of publication.

## Case 1

**Fig. 1:**
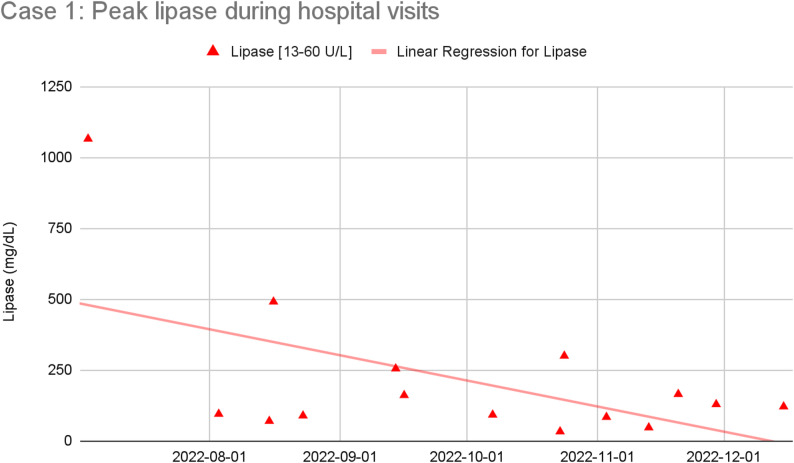
Plot and trendline of peak lipase level obtained during each ED visit or inpatient admission for abdominal pain, showing that the peak lipase level during hospital visits decreased over time

A 24-year-old Haitian male began drinking alcohol as a young teenager and then more heavily three years prior to admissions, due to depression, trauma, and boredom. He had 17 hospital visits for abdominal pain over six months. Early workups revealed elevated lipase and CT findings consistent with acute pancreatitis. He was treated with opioids during each admission and discharged with short courses of oxycodone. He later presented to the ED with abdominal pain that improved rapidly with morphine alone, often with normal lipase levels. Figure 1 shows the decreasing trend in his peak lipase levels over time, suggesting an etiology frequently not consistent with pancreatitis.

He also experienced episodes of precipitated withdrawal from naltrexone for treatment of alcohol use disorder that was started after completion of outpatient opioid treatment, suggesting the development of physiologic opioid dependence. Over time, it became clear that he used small amounts of alcohol to self-treat abdominal pain, with the pain preceding alcohol consumption rather than being the result of it. He described withdrawal symptoms such as diarrhea, anxiety, and relief only with IV opioids. Of note, he did not meet criteria for OUD. The inpatient team initiated buprenorphine during his final admission as a way to address physiologic opioid dependence and prevent repeat episodes of opioid withdrawal. They linked him to outpatient care and recovery support and he has remained in recovery from alcohol and hospitalization-free for over 28 months. He continues daily buprenorphine, has found community and support in recovery meetings, works full-time, and exercises regularly.

## Case 2

A 37-year-old Indian woman with insulin-dependent diabetes, polycystic ovarian syndrome, hypertriglyceridemia, steatohepatitis, and depression had 70 admissions over five years for abdominal pain, a majority of which were diagnosed as pancreatitis, often requiring ICU-admissions for insulin drip to normalize her triglyceride level. She had no history of drug or alcohol use. The flares were attributed to possible genetic factors, medication nonadherence, and dietary issues potentially driven by depression. Admission decisions were often based on lipase levels (typically above 500 U/L), but she was frequently discharged with opioids regardless of lab findings. Over a period of seven months, she filled 23 opioid prescriptions covering 140 of 210 days.

**Fig. 2:**
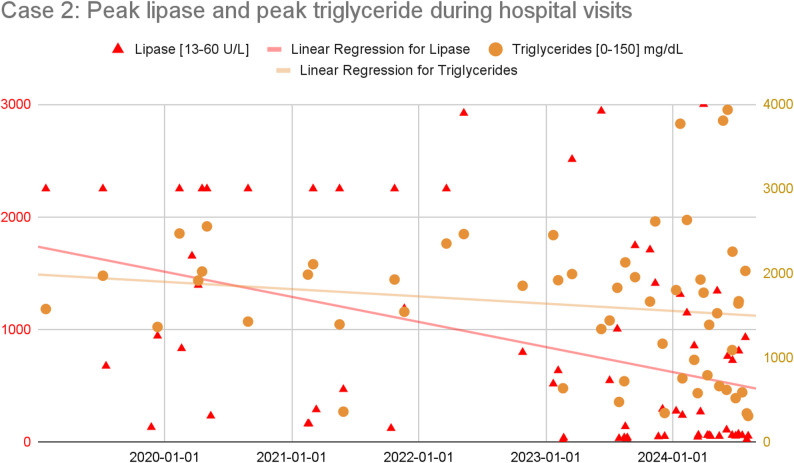
Plot and trendline of peak lipase and peak triglyceride levels obtained during each ED visit or inpatient admission. Due to assay limitation, lipase values above 2250 U/L (respectively, starting early 2022, values above 3000 U/L) are replaced by 2250 U/L (respectively 3000 U/L). The progressive decrease in peak lipase and peak triglyceride during hospital visits suggest that the etiology of the abdominal pain may be changing

**Fig. 3:**
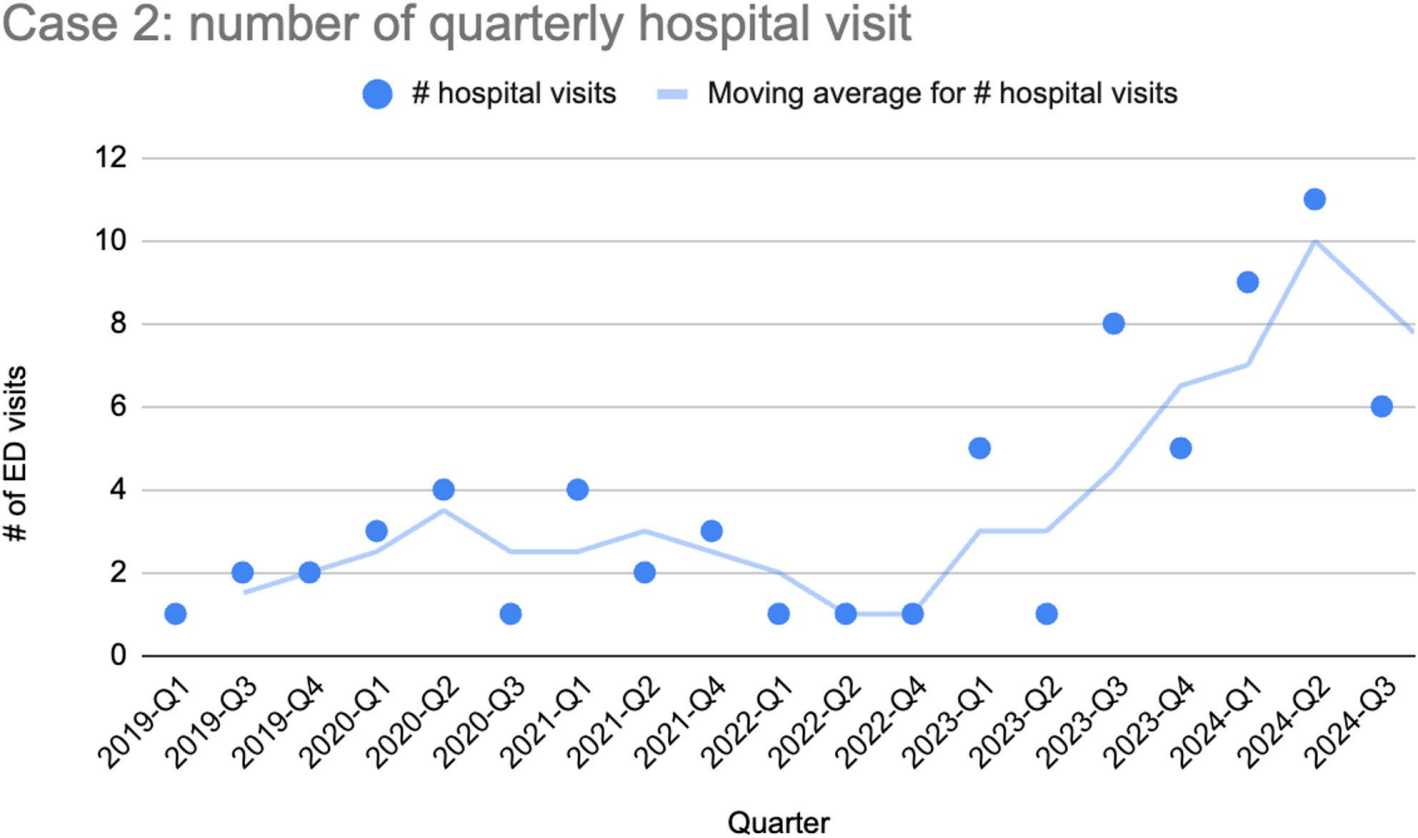
Number of hospital visits per quarter. The number of visits sharply increased starting in 2023

Over her five years of acute presentations, her lipase and triglyceride level values decreased (see Fig. 2) while the frequency of her hospital visits significantly increased (see Fig. 3), suggesting a changing etiology to her presentations.

Eventually, she reported typical opioid withdrawal symptoms — insomnia, anxiety, and sweating — to her primary care provider. Suspecting physiologic opioid dependence, she was started on buprenorphine. Since then, she has had no hospitalizations over 16 months, reports improved self-care, is studying to be a nurse, and continues buprenorphine. She describes the treatment as “life-changing”.

## Discussion

The mainstay of treatment for acute pancreatitis is pain control, often using opioid medication. Recurrent opioid treatment for acute pancreatitis can lead to the development of iatrogenic physiological opioid dependence. Both of our patients initially presented with clear cases of acute pancreatitis but over time their later presentations became more consistent with opioid withdrawal: objective biomarkers of pancreatitis (lipase, triglycerides in the case of triglyceride-induced pancreatitis) decreased while their symptoms rapidly resolved after receiving opioid medication. Once started on maintenance buprenorphine treatment and provided with additional psychosocial support, our patients’ presentations for abdominal pain stopped and they were able to implement behavior changes that likely stabilized the underlying causes of their recurrent episodes of pancreatitis (patient 1 stopped drinking, patient 2 was able to make lifestyle and dietary changes).

It is important for clinicians to have increased awareness of the potential emergence of iatrogenic opioid dependence in patients being treated with opioids for recurrent episodes of pancreatitis. This is particularly relevant given that frequent and similar presentations can lead to the development of anchoring bias and premature diagnostic closure among treating providers [16, 17]. Thus, opioids should be prescribed judiciously during flares and discontinued or quickly tapered down once pain resolves and oral intake resumes. Meta-analyses show no significant day-1 pain difference between patients treated with opioids and those treated with non-opioids like acetaminophen or NSAIDs in cases of mild pancreatitis [18, 19], though further studies are needed to clarify if this applies to moderate to severe pancreatitis flares. Discharge opioids should generally be avoided [20]. 

Some patients will still require use of opioid analgesics and will often be discharged with a short course of them. For patients who require repeat courses of opioid analgesics, physiologic dependence can develop within months of repeated opioid exposure. Since both opioid withdrawal and acute pancreatitis can present with abdominal pain, nausea, and vomiting [21, 22], it is important that clinicians remain vigilant to the signs and symptoms of opioid withdrawal — diarrhea, dilated pupils, goosebumps, yawning, and tremors — often in the context of normal or only mildly elevated lipase levels (i.e., less than 3 times the upper limit of the normal range) that are generally not seen in true pancreatitis [23–25]. In our patients, later admissions featured classic acute pancreatitis flare symptoms but normal lipase and rapid symptom resolution after a single opioid dose — suggesting opioid withdrawal rather than inflammation. For patients who do develop physiological opioid dependence, regardless of their SUD history, transitioning to outpatient buprenorphine could be a beneficial strategy.

Buprenorphine is a partial agonist of the mu opioid receptor and one of three FDA-approved treatments of opioid use disorder. Buprenorphine has a ceiling for respiratory depression, so patients do not overdose on it, and it is recognized as a safer alternative to full opioid agonists [26, 27]. It has also been increasingly acknowledged as an effective treatment for chronic pain [10]. If physiological opioid dependence is established and recognized, even in the absence of opioid use disorder - as was the case for both our patients - it can be successfully addressed with office-based treatment with buprenorphine. When starting buprenorphine, clinicians should be cognizant of associated stigma and potential patient resistance. Clinicians should thus use non-stigmatizing language and frame buprenorphine as a safe, effective treatment that improves quality of life [2]. Educating patients on correct administration is key to minimizing side effects and ensuring success. In some cases, buprenorphine can be tapered off over time, and in others, long-term treatment can be beneficial and help stabilize patients. Additionally, clinicians should take a multimodal approach to managing patients living with chronic pain: utilizing an interdisciplinary team of providers, using non-pharmacologic approaches to pain management, treating mood disorders with medications and therapy, monitoring for and treating substance use disorders, and providing comprehensive primary care with attention to nutrition, exercise, and sleep [12]. 

## Limitations and future directions

This case series represents only two patient experiences in a single hospital system. While our findings offer uncertain generalizability, the challenges we faced in treating our patients is not unique to our setting. While our hospital tries to engage in opioid stewardship, it is possible that more intentional prescribing habits could have prevented opioid dependence from developing. Further research should explore how to minimize opioid dependence and the role of non-opioid medications in adequately treating acute pancreatitis flares. For patients who develop physiological opioid dependence in the absence of opioid use disorder, more research is needed to understand the optimal treatment approach. Specifically when using buprenorphine, it will be important to disentangle the benefits related to treating opioid withdrawal from those related to managing pain. It will also be important to understand whether buprenorphine treatment enables increased patient self-care and ultimately decreases hospitalization rates.

## Conclusion

Physiological opioid dependence resulting from the treatment of recurring pancreatitis, even in the absence of substance use disorder, can result in substantial morbidity. Increased awareness and early recognition of it can help mitigate its medical consequences. Use of buprenorphine for management of iatrogenic opioid dependence is a promising avenue.

## Data Availability

Data sharing is not applicable to this article as no datasets were generated or analyzed during the current study.

## Abbreviations

ED: Emergency Department
NSAIDS: Non-Steroidal Anti-Inflammatory Drugs
